# 489 Nasal-derived Extracellular Vesicles (EVs) carry a cargo of antiviral and immunomodulatory molecules – CORRIGENDUM

**DOI:** 10.1017/cts.2024.687

**Published:** 2025-01-02

**Authors:** Tiziana Corsello, Teodora Ivanciuc, Yue Qu, Antonella Casola, Roberto P Garofalo

The above abstract published with an error in an author name. Tiziana Corsello was incorrectly shown as Tiziana Corsello-Gorgun.

This has been updated in the original abstract.
